# Interleukin-6 suppression reduces tumour self-seeding by circulating tumour cells in a human osteosarcoma nude mouse model

**DOI:** 10.18632/oncotarget.6371

**Published:** 2015-11-09

**Authors:** Yinglong Zhang, Qiong Ma, Tao Liu, Guofeng Guan, Kailiang Zhang, Jiayan Chen, Nan Jia, Shiju Yan, Guanyin Chen, Shiluan Liu, Kuo Jiang, Yao Lu, Yanhua Wen, Haien Zhao, Yong Zhou, Qingyu Fan, Xiuchun Qiu

**Affiliations:** ^1^ Orthopaedic Oncology Institute, Tangdu Hospital, Fourth Military Medical University, Xi'an, Shaanxi, China; ^2^ Department of Microsurgery, Affiliated Hospital of Binzhou Medical University, Binzhou, Shandong, China; ^3^ Department of Orthopedics, No. 88 Hospital of PLA, Tai'an, Shandong, China; ^4^ Department of Occupational and Environmental Health, School of Public Health, Fourth Military Medical University, Xi'an, Shaanxi, China

**Keywords:** osteosarcoma, tumour self-seeding by circulating tumour cells, interleukin-6, metastasis, recurrence

## Abstract

Tumour self-seeding by circulating tumour cells (CTCs) enhances tumour progression and recurrence. Previously, we demonstrated that tumour self-seeding by CTCs occurs in osteosarcoma and revealed that interleukin-6 (IL-6) may promote CTC attraction. Here, we investigated the underlying mechanisms of IL-6 in tumour self-seeding by CTCs. IL-6 suppression inhibited *in vitro* cell proliferation, migration, and invasion. In addition, rhIL-6 activated the Janus-activated kinase/signal transducers and activators of transcription 3 (JAK/STAT3) and mitogen-activated protein kinase/extracellular-signal regulated kinase1/2 (MAPK/ERK1/2) pathways *in vitro*. Both pathways increased cell proliferation, but only the JAK/STAT3 pathway promoted migration. Suppressing IL-6 inhibited *in vivo* tumour growth and metastasis. IL-6 suppression or JAK/STAT3 pathway inhibition reduced CTC seeding in primary tumours. Collectively, IL-6 promotes tumour self-seeding by CTCs in a nude mouse model. This finding may provide a novel strategy for future therapeutic interventions to prevent osteosarcoma progression and recurrence.

## INTRODUCTION

Osteosarcoma is a malignant, highly metastatic cancer occurring primarily in childhood and adolescence. Before the introduction of combination chemotherapy, > 90% of osteosarcoma patients presented with lung metastases [1]. Currently, ∼75% of osteosarcoma patients with non-metastatic disease can be cured [1, 2]. However, the 5-year survival rate of osteosarcoma patients with lung or bone metastases remains approximately 25–50% [2]. The prognosis is worse in patients with recurrence. Their survival rate is < 20%, and there are no well-defined second-line chemotherapeutics [1, 3, 4]. Therefore, metastasis and recurrence are difficult problems for osteosarcoma treatment.

A major cause of tumour recurrence is the increasing number of circulating tumour cells (CTCs), and some chemotherapy-resistant CTCs may become the origin of relapse after treatment [5]. Tumour self-seeding by CTCs supplements the traditional theory of metastasis, which emphasizes the unidirectional movement of CTCs from a primary tumour into the bloodstream followed by seeding distant organs to form metastases [6]. Tumour self-seeding involves the circulation of CTCs back to the primary tumours, promoting primary tumour growth and disease progression [7]. The bidirectional circulation and seeding of CTCs provides a novel explanation for tumour recurrence in postoperative patients. Recurrence may result from CTCs re-seeding local sites with minimal residual tumour cells or into the remaining tumour stroma [8]. Tumour self-seeding by CTCs has been verified in breast cancer, colon cancer, and melanoma [7, 9]. Furthermore, we demonstrated that it also occurs in osteosarcoma [10]. Cytokines such as interleukin 6 (IL-6), which is secreted by osteosarcoma cells, promote CTC recruitment and seeding [10]. In this study, we explored the effects and mechanisms of IL-6 in tumour self-seeding.

IL-6 is secreted not only by T-cells, monocytes, and fibroblasts but also by breast, prostate, and lung cancer cells [11]. IL-6 stimulates the differentiation of B-cells and promotes antibody production [11, 12]. In cancer development, IL-6 promotes proliferation, angiogenesis, migration, invasion, and metastasis [13]. IL-6 signals through the Janus-activated kinase/signal transducers and activators of transcription 3 (JAK/STAT3), mitogen-activated protein kinase/extracellular-signal regulated kinase1/2 (MAPK/ERK1/2), and phosphatidylinositol 3-kinase/Akt (PI3K/Akt) pathways, all of which are implicated in cancer and metastasis [14].

In this study, we report that IL-6 suppression inhibits *in vitro* and *in vivo* osteosarcoma growth and metastasis. Moreover, we reveal that IL-6 activates the STAT3 and ERK pathways. We use cryptotanshinone and FR180204, inhibitors of the STAT3 and ERK pathways, respectively, to demonstrate that STAT3 increases both migration and proliferation, whereas ERK only increases proliferation. We also show that suppressing IL-6 or inhibiting the STAT3 pathway significantly decreases CTC seeding in primary tumours. In conclusion, IL-6 and the activated STAT3 pathway represent novel targets to inhibit tumour self-seeding by CTCs, which has great potential to inhibit osteosarcoma progression and enhance survival.

## RESULTS

### Human osteosarcoma cell line SOSP-9607 and its sublines F5M2 and F4 express IL-6

**Figure 1:**
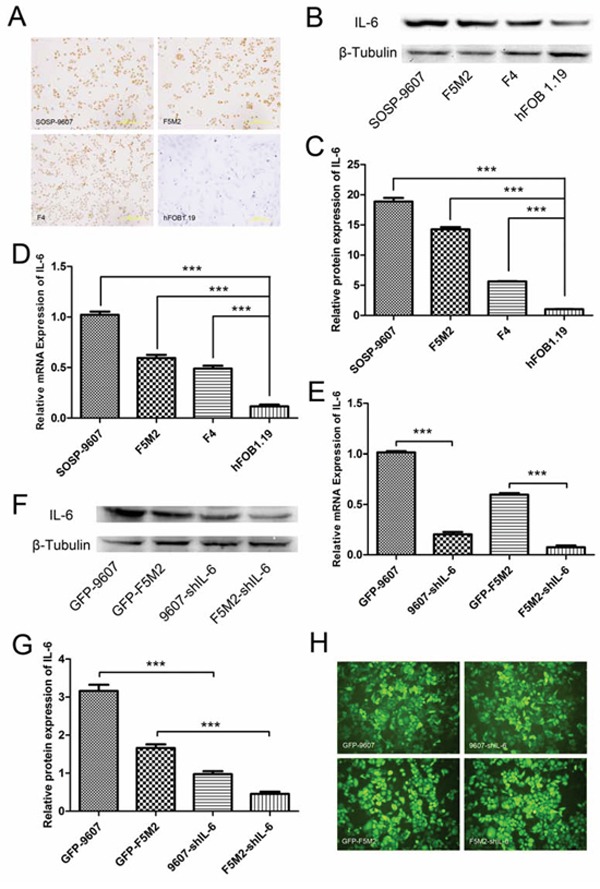
IL-6 expression in osteosarcoma cells and shRNA-mediated suppression **a.** IL-6 expression in SOSP-9607, F5M2, and F4 osteosarcoma cells and control hFOB1.19 normal human osteoblast cells detected by immunocytochemistry (200×). **b.** Western blotting showing IL-6 protein expression in SOSP-9607, F5M2, and F4 human osteosarcoma cells and control hFOB1.19 normal human osteoblast cells. **c.** Relative IL-6 protein expression levels as analysed by ImageJ software. All osteosarcoma cells expressed more IL-6 than did normal osteoblasts (*P* < 0.001). **d.** qRT-PCR results showing IL-6 mRNA expression in SOSP-9607, F5M2, and F4 human osteosarcoma cells and control hFOB1.19 normal human osteoblast cells. All osteosarcoma cells expressed more IL-6 than did normal osteoblasts (*P* < 0.001). **e.** qRT-PCR results showing IL-6 mRNA levels after shRNA silencing. IL-6 mRNA expression was significantly suppressed by shRNA in both the SOSP-9607 and F5M2 cell lines (*P* < 0.001). **f.** IL-6 protein detection by Western blotting after shRNA silencing. **g.** Relative IL-6 protein expression levels analysed by ImageJ. IL-6 protein expression was effectively suppressed by shRNA in both SOSP-9607 and F5M2 cells (*P* < 0.001). **h.** GFP fluorescence images of GFP-9607, 9607-shIL-6, GFP-F5M2 and F5M2-shIL-6 cells (200×). *P < 0.05, **P < 0.01, ***P < 0.001.

Using IL-6 immunocytochemistry, we found that the human osteosarcoma cell line SOSP-9607 and its sublines F5M2 and F4 expressed IL-6 (Figure 1a). Compared with these osteosarcoma cells, the human osteoblastic cell line hFOB 1.19 expressed almost no IL-6 (Figure 1a). We used Western blotting to confirm that all these cell lines express IL-6. After analysis with ImageJ software, one-way ANOVA and the Newman-Keuls multiple comparison test, we found that osteosarcoma cells (SOSP-9607, F5M2 and F4) expressed higher levels of IL-6 than did normal osteoblastic cells (hFOB 1.19) (*P* < 0.001, Figure 1b and 1c). Quantitative reverse transcription polymerase chain reaction (qRT-PCR) results were consistent with the Western blotting results (Figure 1d).

### Small hairpin RNA (shRNA) significantly suppresses IL-6 expression in human osteosarcoma cells

IL-6 expression was significantly reduced by shRNA in SOSP-9607 and F5M2 cells. qRT-PCR revealed that shRNA-2 reduced IL-6 expression by 79.94% in SOSP-9607 cells, and shRNA-1 reduced its expression by 87.30% in F5M2 cells (Figure 1e). Western blotting against IL-6 protein and data analysis using ImageJ software demonstrated that the shRNAs reduced IL-6 expression by > 70% (SOSP-9607 or F5M2; Figures 1f and 1g). The green fluorescent protein (GFP) images of these cells were provided in Figure 1h.

### IL-6 knockdown reduces the proliferative capacity of human osteosarcoma cells

**Figure 2:**
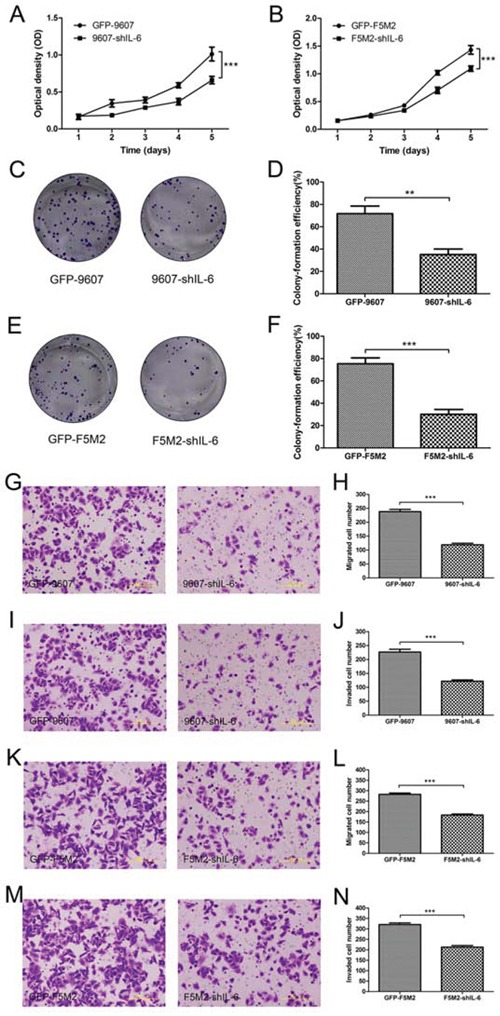
Inhibition of cell proliferation, migration, and invasion after shRNA treatment **a.** MTT assay for the GFP-9607 and 9607-shIL-6 cells. The proliferation rate of the 9607-shIL-6 cells was significantly lower than that of the GFP-9607 cells (*P* < 0.001). **b.** MTT assay for the GFP-F5M2 and F5M2-shIL-6 cells. The proliferation rate of the F5M2-shIL-6 cells was significantly lower than that of the GFP-F5M2 cells (*P* < 0.001) **c.** Representative images of colony formation assays for the GFP-9607 and 9607-shIL-6 cells. **d.** The colony formation efficiency of the 9607-shIL-6 cells was significantly lower than that of GFP-9607 cells (*P* < 0.01). **e.** Representative image of the colony formation assay for the GFP-F5M2 and F5M2-shIL-6 cells. **f.** The colony formation efficiency of the F5M2-shIL-6 cells was significantly lower than that of the GFP-F5M2 cells (*P* < 0.001). **g.** Representative images of the GFP-9607 and 9607-shIL-6 cells in the Transwell migration assay. **h.** The migration rate of the 9607-shIL-6 cells was lower than that of the GFP-9607 cells (*P* < 0.001). **i.** Representative images of the GFP-9607 and 9607-shIL-6 cells in the Transwell invasion assay. **j.** The invasive capacity of the 9607-shIL-6 cells was lower than that of the GFP-9607 cells (*P* < 0.001). **k.** Representative images of the GFP-F5M2 and F5M2-shIL-6 cells in the Transwell migration assay. **l.** The migration rate of the F5M2-shIL-6 cells was lower than that of the GFP-F5M2 cells (*P* < 0.001). **m.** Representative images of the GFP-F5M2 and F5M2-shIL-6 cells in the Transwell invasion assay. **n.** The invasive capacity of the F5M2-shIL-6 cells was lower than that of the GFP-F5M2 cells (*P* < 0.001). *P < 0.05, **P < 0.01, ***P < 0.001.

Using a tetrazolium dye (MTT)-based assay for cell growth, we found that stable IL-6 knockdown reduced the proliferation rates of SOSP-9607 (*P* < 0.001, Figure 2a) and F5M2 cells (*P* < 0.001, Figure 2b) compared to the respective control cells. Colony formation assays demonstrated that stable IL-6 knockdown cells formed fewer colonies than did control cells for both SOSP-9607 (*P* < 0.01, Figures 2c and 2d) and F5M2 cells (*P* < 0.001, Figures 2e and 2f).

### IL-6 knockdown reduces the migration and invasion of human osteosarcoma cells

The number of IL-6 knockdown cells migrating across the inserts in a Transwell migration assay was significantly lower, compared with the control group, for both SOSP-9607 (Figures 2g and 2h, *P* < 0.001) and F5M2 cells (Figures 2k and 2l, *P* < 0.001). These Transwell invasion assay results were consistent with reduced migration in both SOSP-9607 (Figures 2i and 2j, *P* < 0.001) and F5M2 cells (Figures 2m and 2n, *P* < 0.001).

### rhIL-6 stimulates F5M2 cells by activating the JAK/STAT3 and MAPK/ERK1/2 pathways

**Figure 3:**
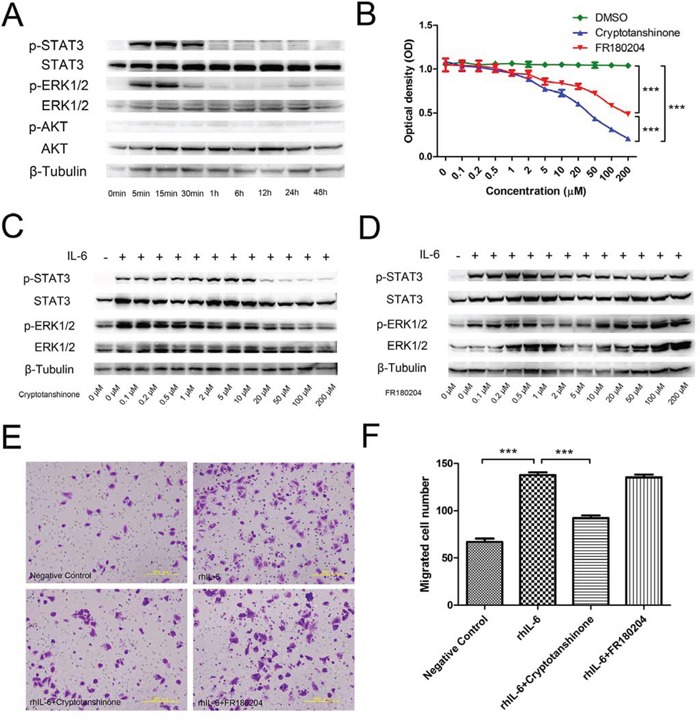
Signalling pathway activation in F5M2 cells after rhIL-6 treatment **a.** Changes in the expression of p-STAT3, p-ERK1/2, p-AKT, STAT3, p-ERK1/2 and AKT according to Western blotting. p-STAT3 and p-ERK1/2 were detected during the first 30 min of rhIL-6 treatment, indicating that rhIL-6 activated the JAK/STAT3 and MAPK/ERK1/2 pathways but not the PI3K/AKT pathway. **b.** MTT assay for F5M2 cells treated with different concentrations of the STAT3 inhibitor cryptotanshinone or the ERK1/2 inhibitor FR180204. Both inhibitors reduced F5M2 cell proliferation compared with the control DMSO (*P* < 0.001). Cryptotanshinone had a substantially greater inhibitory effect than FR180204 (*P* < 0.001). ED50_cryptotanshinone_=19.03 μM and ED50_FR180204_=89.20 μM. **c.** Pre-treatment of F5M2 cells with 50 μM cryptotanshinone prior to rhIL-6 incubation effectively reduced p-STAT3 levels. **d.** Pre-treatment of F5M2 cells with 2 μM FR180204 prior to rhIL-6 incubation effectively reduced p-ERK1/2 expression. **e.** Representative images of F5M2 cells with or without inhibitor treatment before rhIL-6 incubation in the Transwell migration assay. **f.** Cryptotanshinone but not FR180204 significantly inhibited cell migration by rhIL-6 (*P* < 0.001). *P < 0.05, **P < 0.01, ***P < 0.001.

We measured the changes in the phosphorylation of the JAK/STAT3, MAPK/ERK1/2, and PI3K/Akt pathways (Figure 3a). After rhIL-6 treatment, p-STAT3 increased and remained phosphorylated at 5 min, 15 min, and 30 min. The phosphorylation then disappeared. p-ERK1/2 increased at 5 min and 15 min, but the phosphorylation nearly disappeared at 30 min and then completely disappeared. p-AKT levels did not change and exhibited almost no phosphorylation. The expression of total STAT3, total ERK1/2, total AKT and the internal control β-tubulin was consistent throughout.

### STAT3 and ERK1/2 inhibitors inhibit the proliferation of F5M2 cells

After treating F5M2 cells with different concentrations of the STAT3 inhibitor cryptotanshinone or the ERK1/2 inhibitor FR180204 for 24 h, we measured the optical density using an MTT assay (Figure 3b). The absorbance of the inhibitor groups was reduced as the inhibitor concentration increased (*P* < 0.001). The control DMSO group exhibited no significant changes (*P* > 0.05). Both inhibitors reduced the proliferation of F5M2 cells compared with the DMSO controls (*P* < 0.001). Cryptotanshinone had a substantially higher inhibitory effect on proliferation than did FR180204 (*P* < 0.001). Using the ED50 calculation software, we found that cryptotanshinone had an ED50 of 19.03 μM and FR180204 had an ED50 of 89.20 μM.

### The STAT3 inhibitor cryptotanshinone inhibits the migration of F5M2 cells

Using Western blotting and ImageJ software analysis, we found that treatment with either 50 μM cryptotanshinone or 2 μM FR180204 reduced the expression of p-STAT3 or p-ERK1/2, respectively, by approximately the same degree (more than 70%) (Figure 3c and 3d). Therefore, we used 50 μM cryptotanshinone and 2 μM FR180204 for subsequent assays because of their similar abilities to inhibit phosphorylation. Using the Transwell migration assay (Figures 3e and 3f), we found that the number of migrated cells in the rhIL-6-treated group was higher than in the negative control group (*P* < 0.001). Moreover, the number of migrated cells in the rhIL-6- and cryptotanshinone-treated group was substantially lower than in the rhIL-6-treated group (*P* < 0.001), but the number in the rhIL-6- and FR180204-treated group was not significantly different (*P* > 0.05).

### IL-6 shRNA suppresses the growth and metastasis of primary tumours in nude mouse models

**Figure 4:**
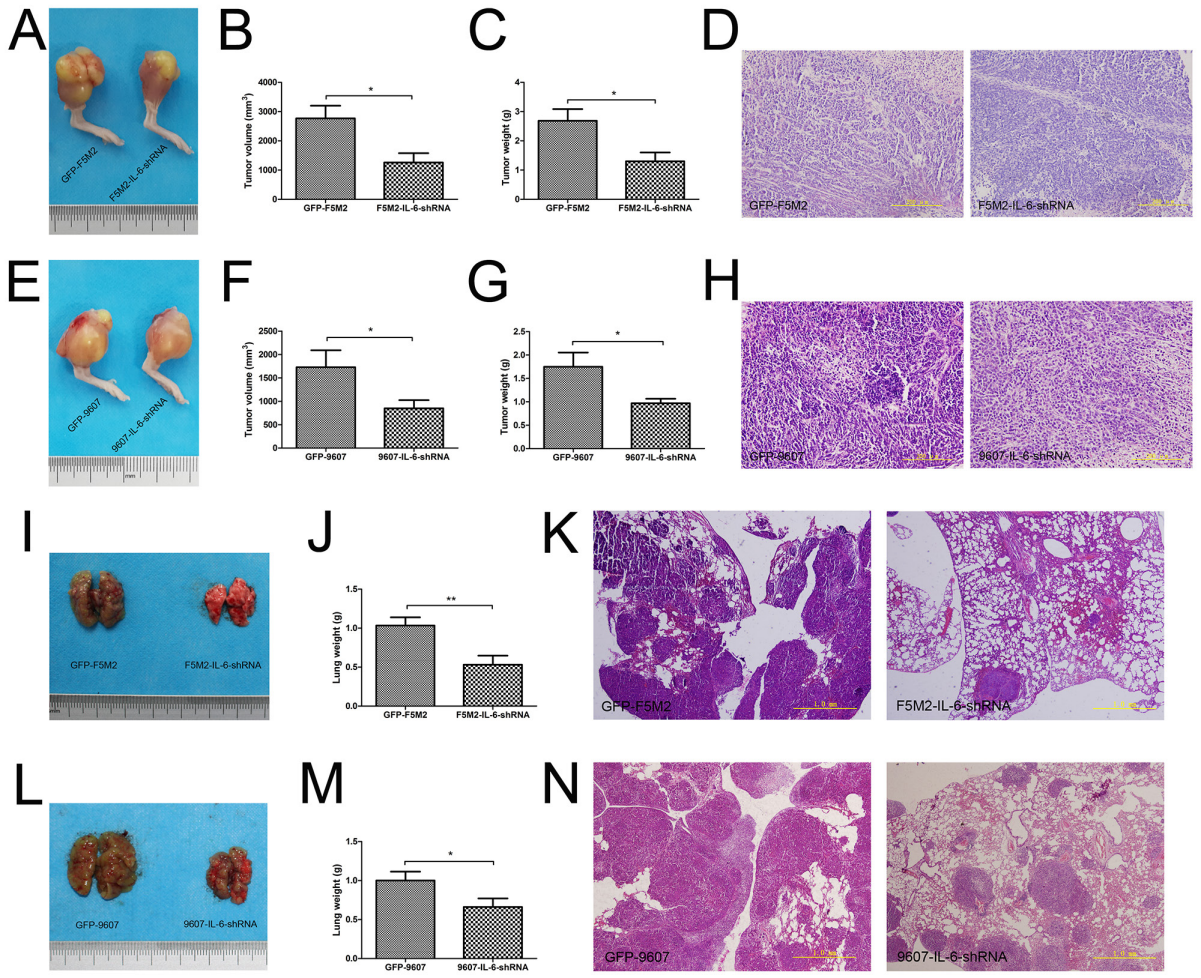
IL-6 shRNA suppresses the growth and metastasis of primary tumours in nude mouse models **a.** Representative images of primary GFP-F5M2 and F5M2-shIL-6 tumours in nude mouse models. **b.** The tumour volumes of the F5M2-shIL-6 group were smaller than those of the control GFP-F5M2 group (*P* < 0.05). **c.** The tumour weights of the F5M2-shIL-6 group were lower than those of the control GFP-F5M2 group (*P* < 0.05). **d.** Representative H&E-stained paraffin-embedded sections of primary tumours from the GFP-F5M2 and F5M2-shIL-6 groups (200×). **e.** Representative images of primary GFP-9607 and 9607-shIL-6 tumours in nude mouse models. **f.** The tumour volumes of the 9607-shIL-6 group were smaller than those of the control GFP-9607 group (*P* < 0.05). **g.** The tumour weights of the 9607-shIL-6 group were less than those of the control GFP-9607 group (*P* < 0.05). **h.** Representative images of H&E-stained paraffin-embedded sections of the primary GFP-9607 and 9607-shIL-6 tumours (200×). **i.** Representative images of lung metastases from GFP-F5M2 and F5M2-shIL-6 cells in nude mouse models. **j.** The lung weights of the F5M2-shIL-6 group were less than those of the control GFP-F5M2 group (*P* < 0.01). **k.** Representative images of H&E-stained paraffin-embedded sections of lungs from the GFP-F5M2 and F5M2-shIL-6 groups (200×). **l.** Representative images of lung metastases from the GFP-9607 and 9607-shIL-6 cells in nude mouse models. **m.** The lung weights of the 9607-shIL-6 group were less than those of the control GFP-9607 group (*P* < 0.05). **n.** Representative images of H&E-stained paraffin-embedded lung sections from the GFP-9607 and 9607-shIL-6 groups (200×). *P < 0.05, **P < 0.01.

We established primary tumours using osteosarcoma cells and their IL-6 knockdown counterparts. The F5M2-shIL-6 tumours grew more slowly than did the control GFP-labelled F5M2 (GFP-F5M2) tumours (Figure 4a) based on tumour volumes (Figure 4b, *P* < 0.05) and weights (Figure 4c, *P* < 0.05). Haematoxylin-eosin (H&E) staining identified the tumour tissues (Figure 4d). We observed a similar tendency in the SOSP-9607 group (Figure 4e) based on tumour volumes (Figure 4f, *P* < 0.05), weights (Figure 4g, *P* < 0.05) and H&E staining (Figure 4h). Lung weights were substantially greater in the control groups than in the IL-6 knockdown groups for both F5M2 (Figures 4i and 4j) and SOSP-9607 cells (Figures 4l and 4m). We observed fewer metastases in H&E-stained lung tissues in the IL-6 knockdown groups compared with the control groups (Figures 4k and 4n).

### F5M2 cells have a substantially higher tumour self-seeding ability than F4 cells

**Figure 5:**
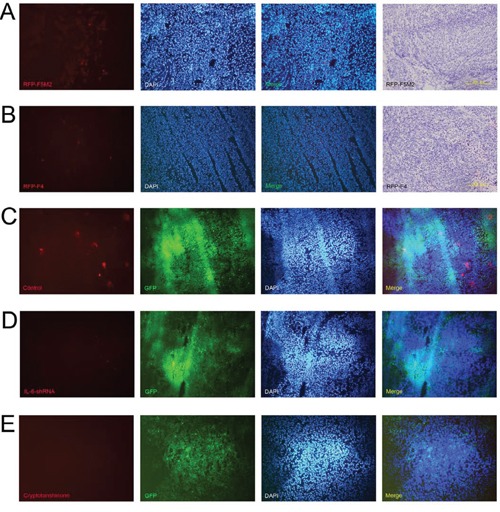
IL-6 shRNA and cryptotanshinone both inhibit the seeding of CTCs into primary tumours Representative fluorescence images from frozen sections of the primary tumour of a tumour self-seeding nude mouse model established using RFP-F5M2 cells **a.** or RFP-F4 cells **b.** as CTCs. F5M2 cells have a substantially higher seeding ability than F4 cells in the tumour self-seeding nude mouse model. The nuclei were counterstained with 4′,6-diamidino-2-phenylindole (DAPI), and the red and blue fluorescence images were merged. The frozen sections were then subjected to H&E staining after fluorescence observation (200×). **c.** Representative fluorescence images of frozen sections of a GFP-9607 primary tumour from a tumour self-seeding nude mouse model established using RFP-F5M2 cells as CTCs. **d.** Representative fluorescence image of a frozen section of a 9607-shIL-6 primary tumour from a tumour self-seeding nude mouse model established using RFP-F5M2 cells as CTCs. **e.** Representative fluorescence images of frozen sections of a GFP-9607 primary tumour from a tumour self-seeding nude mouse model treated with cryptotanshinone and established using RFP-F5M2 cells as CTCs. All of the sections (c, d and e) were counterstained with DAPI, and the red, green and blue fluorescence images were merged. The IL-6 shRNA- and cryptotanshinone-treated groups displayed inhibition of CTC seeding compared with the control groups.

After establishing primary tumours with the SOSP-9607 cell line, we established tumour self-seeding models using RFP-labelled F5M2 or F4 (RFP-F5M2 or RFP-F4) cells as CTCs. RFP-F5M2 cells seeded the primary tumour substantially more frequently than did RFP-F4 cells (Figures 5a and 5b).

### Both IL-6 shRNA and cryptotanshinone inhibit tumour self-seeding by CTCs

After establishing primary tumours with 9607-shIL-6 cells or control GFP-9607 cells in tumour self-seeding models, the seeding of RFP-F5M2 cells (CTCs) was significantly reduced in the IL-6 shRNA group compared with the control group (Figures 5c and 5d). We then established an additional group that underwent cryptotanshinone treatment. Here, the RFP-F5M2 cell seeding was also significantly reduced compared with the control group (Figures 5c and 5e).

### High IL-6 expression significantly correlates with postoperative recurrence in clinical osteosarcoma specimens

**Figure 6:**
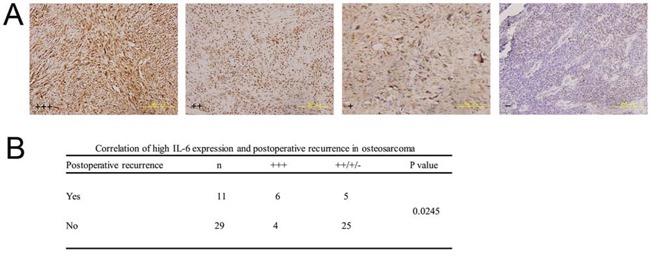
Correlation between high IL-6 expression and postoperative recurrence in clinical osteosarcoma specimens **a.** Representative images of paraffin-embedded sections of clinical osteosarcoma specimens with IL-6 +++, ++, +, and – expression by immunohistochemistry (200×). All sections were counterstained with haematoxylin solution. **b.** High IL-6 expression (+++) was closely correlated with postoperative recurrence in osteosarcoma specimens using the Chi-square test with Yates' correction (*P* < 0.05).

We evaluated IL-6 immunohistochemical staining in clinical osteosarcoma specimens from 11 patients with postoperative recurrence and 29 patients with a first operation. High IL-6 expression significantly correlated with postoperative recurrence in clinical osteosarcoma specimens (Figure 6, *P* < 0.05).

## DISCUSSION

Osteosarcoma treatment is primarily surgery with adjuvant chemotherapy, and the cure rate of patients with simple primary tumours is approximately 70%. However, postoperative recurrence remains a complex problem [1, 3]. Many osteosarcoma patients have high levels of CTCs post-operation [15]. Tumour self-seeding by CTCs provides a novel explanation for osteosarcoma recurrence. Our previous study demonstrated that tumour self-seeding occurs in osteosarcoma nude mouse models and that IL-6 might accelerate this process [10]. This study determined that IL-6 is a key factor promoting self-seeding and explored the underlying mechanism.

To investigate the role of IL-6 in tumour self-seeding, we reduced IL-6 levels in primary osteosarcoma cells. We previously demonstrated that primary osteosarcoma cells (SOSP-9607) secreted more IL-6 than did CTCs (F5M2 or F4 cells) [10]. In this study, we measured IL-6 expression in tumour cells and found that all osteosarcoma cells expressed IL-6. SOSP-9607 cells expressed substantially higher levels, consistent with previous findings [10] (Figures 1a–1d). We then knocked down IL-6 using shRNA to determine whether the loss of IL-6 inhibited CTC attraction to primary tumours. We established stable IL-6 knockdown cell lines using IL-6 shRNA (Figures 1f–1h) and investigated the changes in proliferation, migration, and invasion of the shIL-6 cell lines compared with their respective controls. Using MTT and cell colony formation assays, we determined that IL-6 knockdown suppressed the proliferation of osteosarcoma cells (Figures 2a–2f). Moreover, Transwell migration and invasion assays showed that IL-6 knockdown decreased both the migration and invasion of osteosarcoma cells *in vitro* (Figures 2g–2n). Thus, IL-6 increased the proliferation, migration, and invasion of osteosarcoma cells *in vitro*. Mesenchymal stem cells (MSCs) promote osteosarcoma proliferation and metastasis by secreting IL-6 [16]. Compared with MSCs, which were limited in number, primary tumour cells were more numerous in the tumour mass. Our study further demonstrated that IL-6 expressed by primary osteosarcoma cells had a stronger effect in promoting growth and metastasis.

In our previous study, we revealed that rhIL-6 had an attractive effect on F5M2 cells, which could make CTC seeding more efficient [10]. To further explore the underlying mechanisms of the CTC attraction of IL-6, we chose rhIL-6 and the CTC substitute F5M2 cells to simulate *in vivo* conditions. We investigated three well-known pathways that might be associated with this process and found that the JAK/STAT3 and MAPK/ERK1/2 pathways were activated (Figure 3a). IL-6 induces STAT3 activation, and persistently activated STAT3 promotes the proliferation and metastasis of many cancers [16]. STAT3 is a novel cancer drug target, and many inhibitors are being developed [17]. Cryptotanshinone, extracted from *Salvia miltiorrhiza* Bunge, binds directly to the SH2 domain of STAT3, inhibiting STAT3 phosphorylation and dimerization [18]. Cryptotanshinone inhibits growth and induces apoptosis in prostate cancer cells [18, 19] and breast cancer cells [20, 21]. IL-6 also activated the MAPK/ERK pathway. Activated ERK is involved in tumour growth, angiogenesis, apoptosis, and drug resistance in many cancers [22–25], and ERK inhibition is a potential cancer treatment [26]. FR180204, a selective inhibitor of ERK, competitively disturbs the ability of ATP to bind ERK, blocking protein interactions in the ERK pathway [27]. FR180204 inhibits the proliferation, growth and survival of certain tumour cells [28, 29].

We used different concentrations of the two inhibitors with the MTT test to reveal their effects on osteosarcoma cell proliferation. Figure 3b shows that both cryptotanshinone and FR180204 suppressed tumour cell proliferation, indicating that both pathways are involved in osteosarcoma cell proliferation. Cryptotanshinone was a more potent inhibitor of proliferation than was FR180204. To identify the pathway associated with increased migration, we used Transwell migration assays with or without inhibitor treatment. The JAK/STAT3 pathway was responsible for the majority of the IL-6 effect on cell migration (Figures 3e–3f). Therefore, we speculated that the activation of the JAK/STAT3 pathway might promote the CTC attraction during tumour self-seeding. Activation of the JAK/STAT3 pathway enhances tumour progression, proliferation, invasion, immunosuppression, and therapy resistance [30, 31]. Our study aimed to further explore its role in promoting tumour self-seeding by CTCs.

In animal experiments, we investigated the effect of knocking down IL-6 expression in nude mouse models. Suppressing IL-6 significantly reduced growth and metastasis in both SOSP-9607 and F5M2 nude mouse primary tumour models (Figure 4), in accordance with the *in vitro* results (Figure 2). Therefore, we concluded that IL-6 promoted tumour development. Subsequently, we used the F5M2 subline as the CTCs in the tumour self-seeding model instead of the F4 subline used in our previous study [10] because F5M2 cells seed more efficiently (Figures 5a and 5b). We established tumour self-seeding models with or without shRNA or inhibitor treatment and found that both IL-6 shRNA and cryptotanshinone inhibited the seeding of RFP-labelled CTCs into primary tumours (Figures 5c–5e). Therefore, IL-6 and its activated STAT3 pathway may represent effective targets for blocking tumour self-seeding by CTCs.

To further investigate the correlation between IL-6 and tumour self-seeding in clinical osteosarcoma patients, we evaluated IL-6 immunohistochemistry in specimens from patients with postoperative recurrence and from patients undergoing a first operation. Tumour self-seeding by CTCs might partially explain postoperative recurrence, as residual tumour foci or oedematous tissues surrounding the tumour might attract CTCs from the bloodstream, re-seed the local area and eventually cause or worsen recurrence [7, 8]. The strongly positive IL-6 expression was significantly higher in patients with postoperative recurrence than in control patients (Figure 6). Thus, high IL-6 expression closely correlated with postoperative recurrence in osteosarcoma patients. IL-6 might promote tumour self-seeding by CTCs in clinical situations.

In conclusion, IL-6 enhances tumour self-seeding by CTCs. IL-6 and the activated JAK/STAT3 pathway may serve as efficient targets for blocking or suppressing tumour self-seeding. Moreover, the STAT3 inhibitor cryptotanshinone can potentially be used to delay tumour progression and improve the osteosarcoma survival rate. Further research into tumour self-seeding by CTCs in osteosarcoma will lead to more specific, comprehensive treatments.

## MATERIALS AND METHODS

### Cell culture

The human osteosarcoma cell line SOSP-9607 was established and maintained by our institute. F5M2 and F4 sublines with different metastatic potentials were isolated from the SOSP-9607 cell line [32, 33]. F5M2 and F4 cells were labelled with red fluorescent protein (RFP) by Dr. Yinglong Zhang and Prof. Shi Ke [10]. All cells were cultured in RPMI-1640 culture medium (HyClone, USA) supplemented with 10% foetal bovine serum (FBS) (HyClone, USA) and maintained at 37°C with 5% CO_2_. The human osteoblastic cell line hFOB 1.19 purchased from the Type Culture Collection of the Chinese Academy of Sciences (Shanghai, China) was maintained in DMEM/F12 culture medium (Gibco, USA) supplemented with 10% FBS (PAA, Germany), L-glutamine (150 mg/L, Sigma-Aldrich, USA), NaHCO_3_ (1.5 g/L, Sigma-Aldrich, USA), and G418 (0.3 mg/mL, Sigma-Aldrich, USA) at 33.5°C.

### Immunocytochemistry for IL-6

Cells were seeded onto glass coverslips overnight. The slips were then washed with PBS three times and fixed with 4% polyoxymethylene for 10 min. After incubation with Triton X-100 (Sigma-Aldrich, USA) for 20 min and 5% BSA buffer for 1 h, the cells were incubated with a polyclonal human IL-6 antibody (1:200 dilution, GeneTex, Germany). After incubation at 4°C overnight, the cells were washed with PBS three times and then incubated with the secondary antibody for 1 h. After an additional three washes, the cells were incubated with DAB solution for 5 min and then counterstained with haematoxylin. After mounting, the cells were observed under a microscope to evaluate IL-6 expression.

### qRT-PCR

SOSP-9607, F5M2, and F4 cells were lysed with TRIzol reagent (Life Technologies, USA) after washing with cold PBS three times. All samples were sent to Sangon Biotech Company (Shanghai, China) for subsequent experimental procedures. β-Actin was used as the internal control. The primer sequences were as follows: human IL-6 forward: 5′-GGCAGAAAACAACCTGAACCT-3′ and reverse: 5′-CAAACTCCAAAAGACCAGTGATG-3′; human β-actin forward: 5′-TAGTTGCGTTACACCCTTTCTTG-3′ and reverse: 5′-TCACCTTCACCGTTCCAGTTT-3′. Each experiment was repeated in triplicate.

### shRNA targeting IL-6

Three lentiviruses with shRNA targeting human IL-6 were purchased from Hanbio Biotechnology Company (Shanghai, China). Lentivirus shRNA-1 (CAGCCCTGAGAAAGGAGACATGTAA) was used to knock down IL-6 expression in F5M2 cells. Lentivirus shRNA-2 (GGATTCAATGAGGAGACTTGC) was used to knock down IL-6 expression in SOSP-9607 cells. Knockdown efficiency was assessed by qRT-PCR and Western blotting. Stable IL-6 knockdown osteosarcoma cells (9607-shIL-6 cells or F5M2-shIL-6 cells) and their respective scramble shRNA controls (GFP-9607 cells or GFP-F5M2 cells) were used for subsequent experiments.

### MTT assay

GFP-9607, 9607-shIL-6, GFP-F5M2 and F5M2-shIL-6 cells were seeded into five 96-well plates overnight. The next day, one plate was removed from the incubator, and 20 μl of MTT solution (5 mg/mL, Sigma-Aldrich, USA) was added to each well, including the blank controls. After a 4-h incubation, 150 μl of DMSO (Sigma-Aldrich, USA) was added to each well. The plate was then shaken and measured with a microplate reader (Thermo Scientific, USA) using a 490/630-nm filter. This same procedure was repeated every 24 h until the last plate was measured. Each experiment was repeated in triplicate.

### Colony formation assay

Two hundred GFP-9607, 9607-shIL-6, GFP-F5M2, or F5M2-shIL-6 cells per well were seeded into 6-well plates in triplicate. After culturing for 10 days, each well was washed three times with PBS, fixed with 4% polyoxymethylene for 10 min, and then stained with 4% crystal violet solution for 5 min. After washing, the colonies in each group were counted and imaged. The colony formation efficiency was calculated as follows: colony formation efficiency = (number of colonies/number of inoculated cells) × 100%. Each experiment was repeated in triplicate.

### Transwell migration and invasion assays

The Transwell migration and invasion assays were performed as described in our previous study [10]. The upper chambers were seeded with 1 × 10^5^ cells, and the lower chambers were filled with complete RPMI-1640 medium. Migration was detected using the Transwell inserts only, and invasion was detected using inserts pre-coated with 50 μl of BD Matrigel™ Basement Membrane Matrix (1:3 dilution, BD Biosciences, USA). The 9607-shIL-6 or F5M2-shIL-6 cells were compared to their respective control cells in the assays. The migration assay was evaluated after 18 h of incubation, and the invasion assay was evaluated after 22 h of incubation. Each experiment was repeated in triplicate.

### Western blotting

Cell lysates were collected using RIPA lysis buffer supplemented with protease and phosphatase inhibitor cocktails (Roche, USA) and prepared for blotting detection as described in our previous study [34]. Then, 30 μg of each sample was separated by electrophoresis and transferred to PVDF membranes, which were blocked with 5% BSA or non-fat dry milk for 1 h at room temperature (RT) and then incubated with primary antibodies at 4°C overnight. IL-6 antibody (GeneTex, USA) was diluted 1:1,000. The p-STAT3, p-ERK1/2, p-AKT, STAT3, ERK1/2 and AKT antibodies (Bioworld, USA) were all diluted 1:500. A β-tubulin (1:2,000 dilution) antibody (Sigma-Aldrich, USA) was used as an internal control. The next day, the membrane was incubated with secondary antibodies (1:40,000) at RT for 1 h after washing with Tris-buffered saline with Tween (TBST) three times for 5 min each. Finally, the membrane was incubated with ECL substrate and peroxide solutions (Genshare Biological, China) and scanned using a Bio-Rad imaging system (USA). Each experiment was repeated in triplicate.

### Cell signalling pathway detection

An aliquot of 1×10^6^ F5M2 cells was seeded into 60-mm dishes overnight. The next day, the media were replaced, and the cells were incubated with 100 ng/ml of rhIL-6 solution for 0 min, 5 min, 15 min, 30 min, 1 h, 6 h, 12 h, 24 h or 48 h. Proteins were collected from different treatment duration groups as described above. Western blotting was used to detect (phosphorylated) p-STAT3, p-ERK1/2 and p-AKT and (total) STAT3, ERK1/2 and AKT to determine pathway activation. Each experiment was repeated in triplicate.

### Inhibitor experiments

First, an MTT assay was performed. The STAT3 inhibitor cryptotanshinone and the ERK1/2 inhibitor FR180204 (Selleck, USA) were diluted to obtain concentrations of 0.1, 0.2, 0.5, 1, 2, 5, 10, 20, 50, 100 and 200 μM, with DMSO as the vehicle control. F5M2 cells were seeded at 3×10^3^ per well in 96-well plates overnight. The culture media were then replaced with the inhibitors or control at different concentrations. After 24 h, all plates were subjected to the MTT assay as described above. The inhibitor efficiency test was then performed. F5M2 cells were seeded at 1×10^6^ cells per 60-mm dish overnight. The next day, the media were replaced with different concentrations of the two inhibitors as described above. After a 4-h incubation and two PBS washes, the media were replaced with a 100 ng/ml rhIL-6 solution. After 15 min of rhIL-6 stimulation, proteins from all the groups were collected for Western blotting. Changes in p-STAT3 and p-ERK1/2 were detected to determine the appropriate concentrations of the two inhibitors. Next, the inhibitor migration test was performed. Appropriate concentrations of the two inhibitors were applied to F5M2 cells for 4 h. The cells were then incubated with 100 ng/ml of rhIL-6 for 24 h. Finally, the cells with or without inhibitor treatment and with or without rhIL-6 treatment were harvested for the Transwell migration assay, which was performed as described above. Each experiment was repeated in triplicate.

### Animal models

Each nude mouse (3–4 weeks old; female, *n* = 20) was inoculated with 3×10^6^ SOSP-9607 cells by injection into the tibial plateau of the right leg to establish the primary tumour. After two weeks, the mice were randomly divided into two groups that were respectively injected with RFP-F5M2 or RFP-F4 cells through the tail vein (1×10^6^ cells per mouse). Two weeks later, primary tumours from each group were dissected and prepared for frozen sections as previously described [10]. The seeding conditions of the RFP-labelled cells were observed under a fluorescence microscope and compared between the two groups. We inoculated 3×10^6^ of the GFP-F5M2, F5M2-shIL-6, GFP-9607, and 9607-shIL-6 cells into nude mice to establish primary osteosarcoma models (*n* = 10). After six weeks, the mice were sacrificed to observe primary tumour growth and lung metastasis. In addition to the macro-comparisons of tumour size (volume or weight) and lung weight, H&E staining of the lungs was performed to observe metastases. Additionally, 3×10^6^ GFP-9607 and 9607-shIL-6 cells were each used to establish primary tumour models, with the GFP-9607 group subdivided into two groups (*n* = 10). One GFP-9607 group was treated with 100 μg of cryptotanshinone daily (five days per week) by intravenous tail injection. The other GFP-9607 group and the 9607-shIL-6 group received equal volumes of saline (*n* = 10). Two weeks later, all three groups received injections of RFP-F5M2 cells as CTCs. After 2–3 weeks, primary tumours from each group were removed and dissected. Frozen sections were prepared to evaluate the seeding conditions.

### Immunohistochemistry

Eleven osteosarcoma patients with postoperative recurrence and 29 patients with a first operation were chosen for immunohistochemical analysis. All specimens were collected in our Tangdu hospital following patient approval. Immunohistochemistry was performed as described [10], and the IL-6 antibody was diluted 1:100. Images were acquired under a microscope at 200×. The immunostaining results were evaluated and analysed as described [34].

### Statistical analyses

All statistical analyses were conducted using GraphPad Prism 5.0 (GraphPad Software Inc., CA, USA). The IL-6 Western blot, qRT-PCR, and inhibitor migration assay data were analysed with a one-way ANOVA test and the Newman-Keuls multiple comparison test for post hoc pair comparisons. The MTT assay data upon IL-6 knockdown were analysed with the Wilcoxon signed-rank test. The MTT assay data for the two different inhibitors were analysed with two-way ANOVA and Bonferroni's post hoc test, and ED50 values were calculated. The immunohistochemical data were analysed using the Chi-square test with Yates' correction. The other data were analysed with a two-tailed Student's *t*-test. All data are presented as the means ± S.E.M., and *P* < 0.05 was considered significant.
